# Visuomotor performance at high altitude in COPD patients. Randomized placebo-controlled trial of acetazolamide

**DOI:** 10.3389/fphys.2022.980755

**Published:** 2022-09-08

**Authors:** P. M. Scheiwiller, M. Furian, A. Buergin, L. C. Mayer, S. R. Schneider, M. Mademilov, M. Lichtblau, L. Muralt, U. Sheraliev, T. M. Sooronbaev, S. Ulrich, K. E. Bloch

**Affiliations:** ^1^ University Hospital of Zurich, Department of Respiratory Medicine, Sleep Disorders Center, Zurich, Switzerland; ^2^ Swiss-Kyrgyz High Altitude Medicine and Research Initiative, Zurich, Switzerland, and Bishkek, Kyrgyz Republic; ^3^ National Center for Cardiology and Internal Medicine, Department of Respiratory Medicine, Bishkek, Kyrgyz Republic

**Keywords:** COPD, altitude, acetazolamide, placebo, visuo-motor task, sleep

## Abstract

**Introduction:** We evaluated whether exposure to high altitude impairs visuomotor learning in lowlanders with chronic obstructive pulmonary disease (COPD) and whether this can be prevented by acetazolamide treatment.

**Methods:** 45 patients with COPD, living <800 m, FEV1 ≥40 to <80%predicted, were randomized to acetazolamide (375 mg/d) or placebo, administered 24h before and during a 2-day stay in a clinic at 3100 m. Visuomotor performance was evaluated with a validated, computer-assisted test (Motor-Task-Manager) at 760 m above sea level (baseline, before starting the study drug), within 4h after arrival at 3100 m and in the morning after one night at 3100 m. Main outcome was the directional error (DE) of cursor movements controlled by the participant via mouse on a computer screen during a target tracking task. Effects of high altitude and acetazolamide on DE during an adaptation phase, immediate recall and post-sleep recall were evaluated by regression analyses. www.ClinicalTrials.gov NCT03165890.

**Results:** In 22 patients receiving placebo, DE at 3100 m increased during adaptation by mean 2.5°, 95%CI 2.2° to 2.7° (*p* < 0.001), during immediate recall by 5.3°, 4.6° to 6.1° (*p* < 0.001), and post-sleep recall by 5.8°, 5.0 to 6.7° (*p* < 0.001), vs. corresponding values at 760 m. In 23 participants receiving acetazolamide, corresponding DE were reduced by −0.3° (−0.6° to 0.1°, *p* = 0.120), −2.7° (−3.7° to −1.6°, *p* < 0.001) and −3.1° (−4.3° to −2.0°, *p* < 0.001), compared to placebo at 3100 m.

**Conclusion:** Lowlanders with COPD travelling to 3100 m experienced altitude-induced impairments in immediate and post-sleep recall of a visuomotor task. Preventive acetazolamide treatment mitigated these undesirable effects.

## Introduction

Chronic obstructive pulmonary disease (COPD) affects at least 210 million people worldwide (WHO, 2007). Considering this high prevalence, many people with COPD are expected among travelers to high altitude areas for work or recreational purposes. COPD does not only affect the lung but is also associated with cardiovascular diseases and cognitive impairment in different domains including motor skills and learning. This has been attributed to increased levels of inflammatory mediators, altered cerebral metabolism and hypoxia-induced neuronal dysfunction and damage (Dodd et al., 2010). Whether exposure to hypoxia during high altitude travel exacerbates cognitive performance decrements in COPD patients has not been studied.

In healthy individuals, exposure to high altitude has subtle negative effects on cognitive performance (Hornbein, 2001; Virués-Ortega et al., 2004; Petrassi et al., 2012; McMorris et al., 2017), with complex tasks affected more than simple tasks (Petrassi et al., 2012). But data are inconsistent and effects of high altitude on motor memory have not been extensively studied. In a study in healthy lowlanders staying for 2 days at 2590 m, we found a negative effect on sleep-dependent visuomotor memory consolidation associated with a reduced synchronization of neuronal activity during sleep (Tesler et al., 2015). Acetazolamide, an unselective carbonic anhydrase inhibitor, acts as a respiratory stimulant by inducing a metabolic acidosis through renal loss of bicarbonate; additionally, acetazolamide induces a tissue acidosis in the brain and chemoreceptors, further enhancing ventilation (Swenson and Hughes, 1993). In healthy mountaineers, acetazolamide may improve oxygenation and stabilize periodic breathing and it is established for prevention and treatment of acute mountain sickness (AMS) (Swenson, 1998; Kayser et al., 2012; Bärtsch and Swenson, 2013). However, as there are controversial data on the effect of acetazolamide on cognitive performance (Wang et al., 2013; Yang et al., 2013; Collier et al., 2016; Phillips et al., 2017) further studies corroborating these findings are required.

As visuomotor performance is essential for many activities during high altitude travel, the purpose of the current trial in patients with COPD was to evaluate (1) whether acute altitude exposure impairs visuomotor performance and (2) whether acetazolamide mitigates these impairments in association with improvement of systemic oxygenation.

## Methods

### Study design and setting

We conducted a randomized, placebo-controlled, double-blind, parallel-design trial in lowlanders with COPD evaluating the effect of a 2-day sojourn at 3100 m and of preventive acetazolamide treatment on visuomotor performance. The study was carried out from June to July 2017 in Kyrgyzstan at the National Center of Cardiology and Internal Medicine in Bishkek (760 m), and at the High-Altitude Clinic Too Ashu (3100 m). Data collection was embedded in a larger project evaluating effectiveness of acetazolamide in preventing high altitude-related adverse health effects in patients with COPD (Furian et al., 2022). Baseline characteristics of participants and data on acute mountain sickness have been published (Furian et al., 2022), but data on visuomotor performance, the focus of the current study, have not been reported yet. Baseline measures without application of the study drug were performed in the Kyrgyz National Center of Cardiology and Internal Medicine in Bishkek, Kyrgyzstan, at 760 m. Subsequently, patients were randomly assigned to either acetazolamide or placebo treatment. One to 3 weeks after baseline measurements, patients travelled from Bishkek to the Too Ashu High Altitude Clinic at 3100 m for altitude measurements. Application of acetazolamide or equally looking placebo was started 24 h before ascent and continued throughout the stay at 3100 m. The study was approved by the local ethics committee of Bishkek, Kyrgyzstan, and participants gave written informed consent. This study was registered on clinicaltrials.gov with the identifier NCT03165890.

### Participants

Kyrgyz lowlanders with COPD living in the Bishkek area (mean altitude 760 m) were invited to participate in the study. Inclusion criteria were 20–75 years of age, both sexes, diagnosed with COPD according to GOLD criteria, grades 2-3 (GOLD Science Committee Members, 2022), and FEV_1_ between 40-80% predicted. They had to be born, raised and currently living at low altitude (<800 m).

We excluded patients with recent COPD exacerbation, hypoxemia at low altitude (oxygen saturation on room air <92% at rest), severe comorbidities such as uncontrolled or unstable cardiovascular disease; previous stroke; obstructive sleep apnea syndrome; renal failure, other diseases that may interfere with protocol compliance; allergy to acetazolamide and other sulfonamides.

### Interventions

Baseline assessments were conducted without administration of the study drug. After baseline assessments at 760 m, participants were randomized to receive treatment with acetazolamide capsules (125 mg) or identically looking placebo capsules, 1 in the morning and 2 in the evening (total daily dose 375 mg) administered by an investigator over the 3-days study duration. After 1 day on treatment at 760 m, patients travelled by minibus within 4–6 h to the Too Ashu High Altitude Clinic at 3100 m and stayed there for 2 days.

### Assessments

#### Visuomotor performance test

**FIGURE 1:**
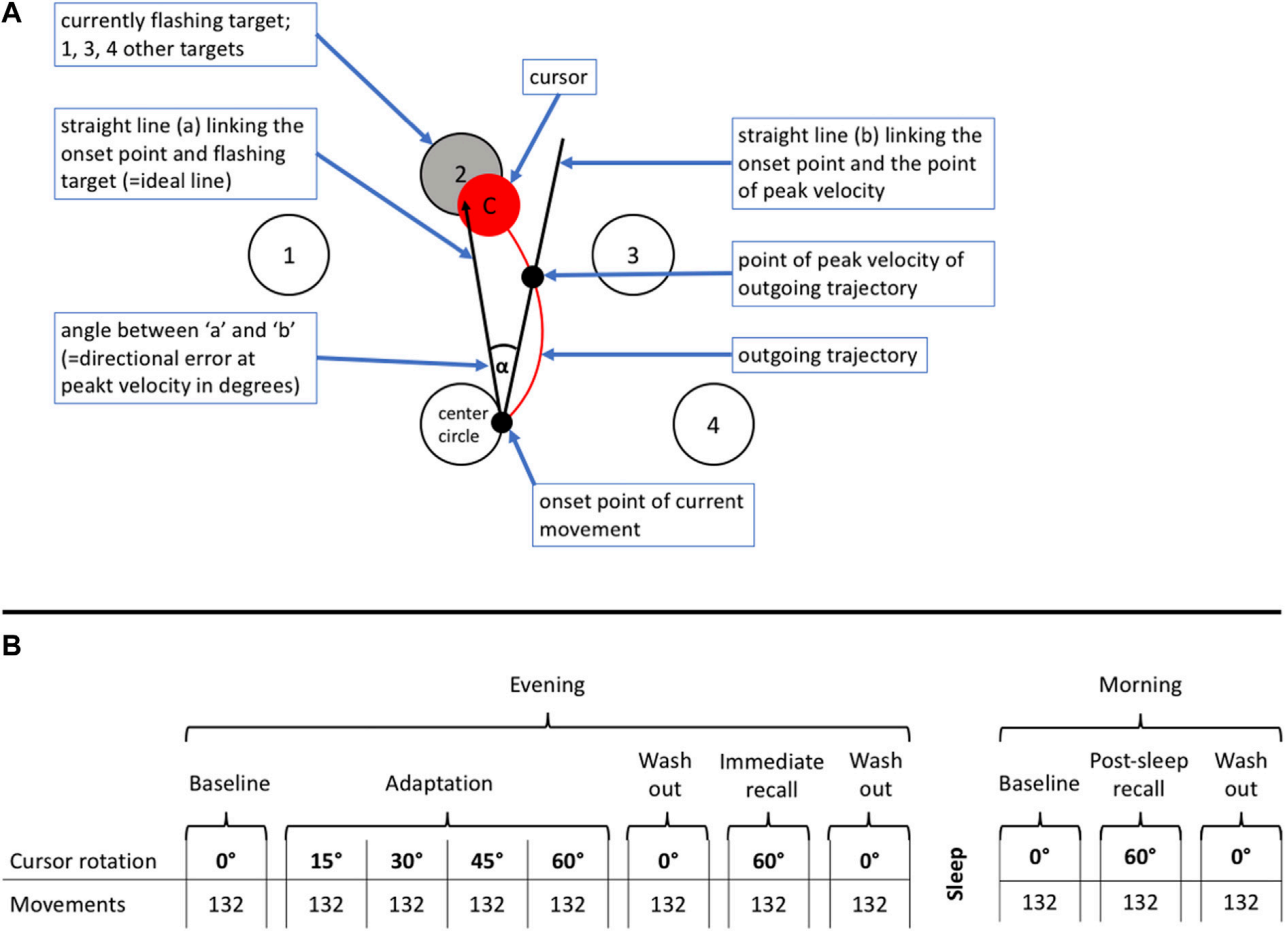
**(A)** Schematic representation of the computer video screen as presented to a patient during the visuomotor learning task. The patients had to operate a handheld mouse on a drawing board below an opaque shield. The position of the mouse was indicated as a cursor on a computer screen. Four different targets (1–4) were located in a semi-circular array around the center. Participants were instructed to move the cursor from the center circle (= starting point) to the flashing target and immediately back, in the most direct and fastest way possible, and in one go. The flashing of targets appeared in a one second interval at a random sequence. **(B)** Explanation of test protocol. The evening part was conducted in the evening on the day of arrival at the study location between 7:00 and 9:00 p.m., the morning part in the following morning between 6:30 and 8:00 a.m. The evening part consisted of an adaptation part followed by an immediate recall. The post-sleep recall in the morning was identical to the immediate recall in the evening. The adaptation part, immediate and post-sleep recall ended with a wash out to reset any residual rotation of the newly acquired motor memory. The test was started without rotation to familiarize the patients with the task. During the adaption part, the rotation increased by steps of 15° after every 132^th^ movement (corresponding 33 movements to each of the four targets) up to a maximum of 60° and ended by a 0° wash-out and followed by the immediate recall, where the rotation switched directly from 0° to 60°. After one night post-sleep recall was assessed in the morning.

We used a previously described visuomotor performance test, the ‘Motor Task Manager’ (ETT S.p.A., Genoa, Italy) (Huber et al., 2004; Landsness et al., 2009). Patients had to operate a handheld mouse to move a cursor from its central position on a computer screen as fast as possible to a target, i.e., one of 4 circles that started flashing at the beginning of a particular test, and back to the center. During target tracking, the software imposed a predefined rotation onto the cursor trajectory, so that the movement direction of the hand differed from that of the cursor. This was unknown to the participants and a shield prevented them from seeing their hand during test execution. Tests were administered in blocks of 132 target presentations. They were started with a baseline block with 0° rotation followed by an adaptation period of 4 successive blocks with progressive increase in cursor rotation from 15° to 60° allowing the participant to learn to compensate cursor rotation as a new visuomotor skill; the adaptation period was completed by a washout with 0° rotation. During subsequent recall periods on the same day and in the following morning, the patient’s ability to execute the newly learnt skill was tested. Tests of immediate recall were presented within minutes after adaptation; post-sleep recall was tested in the following morning to assess overnight memory consolidation. The direction of the initial rotation at 760 m was selected at random. The rotation at 3100 m was imposed in opposite direction to reduce learning bias since adaptation to cursor rotation in one direction does not transfer to the opposite direction (Krakauer and Shadmehr, 2006). Figure 1 illustrates the schematic representation of the computer screen (A) and the test sequences (B).

The three outcomes derived from the Motor Task Manager test were A) the directional error (DE) at peak velocity, defined as the angle between a line from the initial cursor position to the target and a line to the position of the cursor at the peak outward velocity (Figure 1; a lower DE indicates a better performance); B) the reaction time, defined as time elapsed from initiation of target flashing to the start of cursor movement which depends on the time needed to recognize the target and whether the previous movement was timely ended or not; C) trajectory validity, quantified as number of valid observations in percent of total trajectories determined by manually checking every movement for validity according to predefined rules (for example, as too short movement, not in one go, delayed or early onset).

#### Additional assessments

Further assessments included medical history, symptoms of COPD by the COPD Assessment Test (CAT, an 8-item questionnaire with a score-range from 0 to 40, higher scores indicate a subjectively more impaired health status (GOLD Science Committee Members, 2022)). Pulse oximetry and clinical examination, spirometry and arterial blood gas analysis were performed in the morning after the first night at both altitudes.

### Randomization and blinding

Participants were randomized 1:1 to acetazolamide or placebo minimizing for age, sex and COPD severity by a computer algorithm. Participants and investigators were blinded to the drug assignment until conclusion of data analysis.

### Main outcome and sample size estimation

The main outcome was the DE. No separate sample size estimation was performed for the current trial because the sample size was determined by the sample size estimation for the main trial on prevention of high altitude-related adverse health effects into which the current trial was nested (Furian et al., 2022). The minimal important difference in DE and the performance in patients with COPD are unknown. Nevertheless, based on assumptions derived from a similar study (Tesler et al., 2015) in young, healthy individuals, the current study was powered with 92% (alpha 0.05) to detect a 5° difference in DE.

### Statistical analysis

Data for this physiological study were analyzed according to the per protocol principle. A random effects panel data multivariate regression model was applied to assess the effects of high altitude exposure, study drug, age, sex and percentage of valid observations per patient on DE. An interaction term was used between the two predictors altitude and drug. With correcting for valid observations, it was aimed to correct for the fact that all patients performed tests for a second time at 3100 m, where they had fewer invalid movements.

In a further analysis, we replaced the interaction term of altitude and drug with the interaction of pulse oximetry (SpO_2_ measured before conducting the MTM test) and drug to assess if DE depended on SpO_2_. Assuming lower SpO_2_ at the higher altitude, SpO_2_ was coded as a binary variable, 1 representing the upper half of SpO_2_ values and 2 representing the lower half. Conversion of SpO_2_ from a continuous to a binary variable was done for easier interpretation of the regression model.

Corresponding regression models were used to evaluate effects of acetazolamide and high altitude on reaction time and pulse oximetry.

Statistical analyses were conducted using R (R Core Team, 2018) with the plm package (Croissant and Millo, 2008) for panel data multivariable regression analysis.

## Results

### Patient characteristics

**FIGURE 2:**
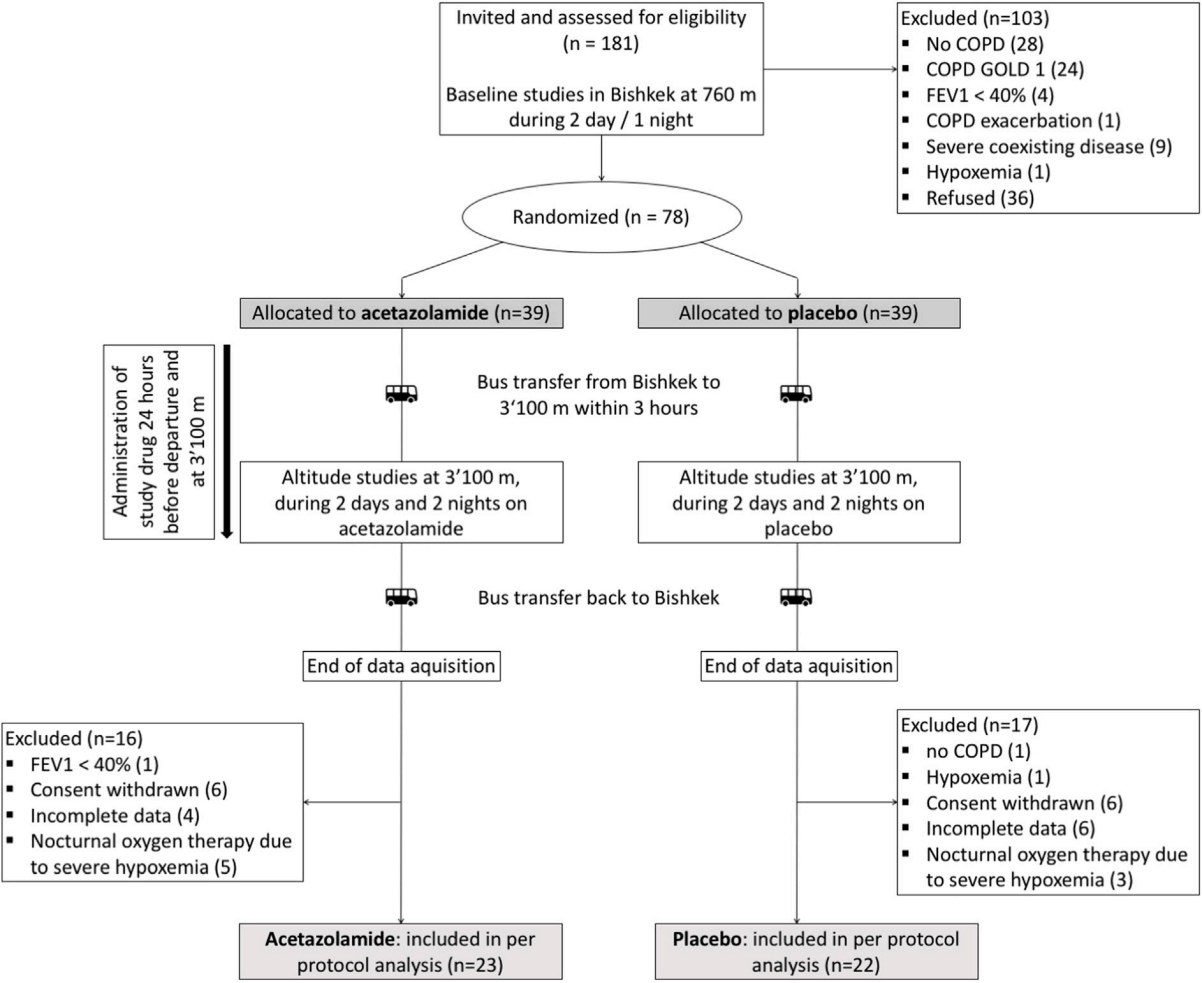
Patient flow.

A total of 78 patients were randomized, 33 had to be excluded for different reasons (Figure 2). Data from 45 patients were included into the per protocol analysis, 22 in the placebo and 23 in the acetazolamide group. Patient characteristics at baseline are described in Table 1. The median age was 59 (range: 35–70) years. 6 women (13%) participated. Median (interquartile range) predicted FEV_1_ was 66.7% (62.4; 71.6) in the acetazolamide group and 70.6% (58.6; 77.5) in the placebo group.

**TABLE 1:** Baseline characteristics of patients (per protocol analysis).

Characteristics	All (*n* = 45)	Acetazolamide (*n* = 23)	Placebo (*n* = 22)
Age (years)	59 (54; 62)	59 (53; 61)	59 (56; 66)
Female sex—no. (%)	6 (13%)	3 (13%)	3 (13%)
Body mass index (kg*m^−2^)	25.7 (24.3; 28.3)	26.7 (25.1; 28.1)	24.9 (23.7; 28.2)
FEV_1_ (% pred)	67.9 (60.5; 75.8)	66.7 (62.4; 71.6)	70.6 (58.6; 77.5)
FEV_1_/FVC (%)	63.4 (55.0; 65.8)	63.4 (58.9; 65.7)	61.7 (54.2; 66.4)
CAT score	9 (6; 11)	9 (5; 11)	9 (7; 11)
Smoking pack-years	24 (4.5; 35)	20 (1; 32)	30 (15; 40)
Ever smoker—no. (%)	35 (78%)	17 (74%)	18 (82%)
SpO_2_ (%)	96 (95; 96)	96 (95; 96)	96 (95; 97)
PaO_2_ (mmHg)	70.3 (65.5; 73.2)	69.0 (65.5; 72.7)	70.9 (66.7; 73.7)
pH	7.41 (7.39; 7.42)	7.40 (7.39; 7.41)	7.41 (7.39; 7.42)
PaCO_2_ (mmHg)	40.2 (37.7; 42.2)	41.6 (38.3; 43.1)	39.3 (37.6; 41.3)
Medication use—no. (%)			
Inhalation therapy (LAMA, LABA)	4 (9%)	3 (13%)	1 (5%)
Antihypertensive medication	4 (9%)	3 (13%	1 (5%)
Platelet aggregation inhibitor	4 (9%)	2 (9%)	2 (9%)
Randomization to CW at Baseline—no. (%)	21 (47%)	9 (39%)	12 (55%)

Values are medians (interquartile range) assessed at 760 m. FEV_1_, forced expiratory volume in the first second of expiration; FVC, forced vital capacity; CAT, COPD assessment test; SpO_2_, pulse oximetry; PaCO_2_, arterial carbon dioxide partial pressure; pH, potential of hydrogen measured by arterial blood sampling; PaO_2_, arterial oxygen partial pressure; LAMA, long acting muscarinergic antagonist; LABA, long acting beta-2-receptor agonist; CW, Clockwise

### Main outcomes: Directional error at peak velocity

**FIGURE 3:**
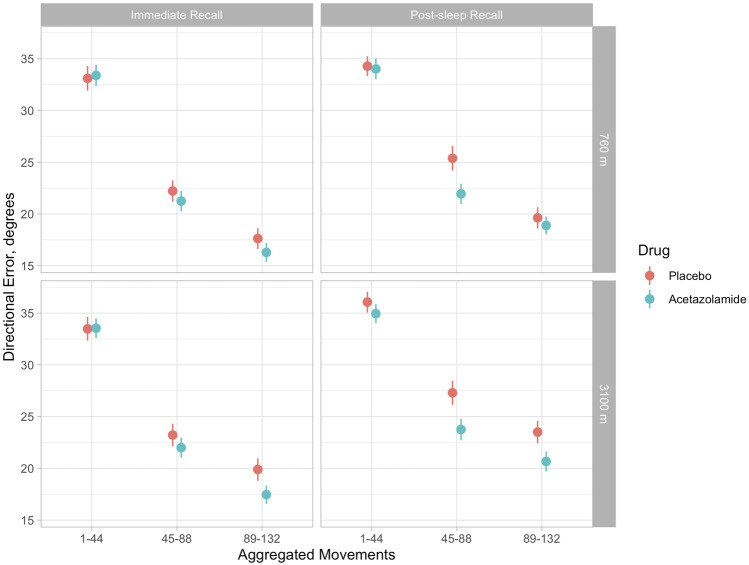
Illustration of means (and 95% confidence intervals) of directional errors for the acetazolamide (blue) and placebo (orange) group in 3 successive blocks of 44 movements each during immediate (evening) and post-sleep (morning) recall tests. Upper panels: baseline evaluations at 760 m were performed before starting the study drug. Upper left panel: immediate recall at 760 m; upper right panel, post-sleep recall at 760 m; lower left panel, immediate recall at 3100 m; lower right panel, post-sleep recall at 3100 m. According to Table 2, in tests at 3100 m, directional errors were significantly reduced by acetazolamide during immediate recall (by 2.7°, 95% CI 1.6–3.7) and post-sleep recall by 3.1°, 95% CI 2.0–4.3). The progressive decrease in directional error with increasing number of the 132 blocks is consistent with a learning effect, while the last block of aggregated movements (89–132) is a surrogate of final performance level.

Multivariate regression analysis showed a significant deterioration (increase) of DE at 3100 m compared to 760 m in the placebo group during adaptation, immediate and post-sleep recall (*p* < 0.001, all instances, Table 2 and Figure 3). High altitude-induced performance decrements were more prominent in immediate and post-sleep recall blocks (5.3°, 95% confidence interval 4.6° to 6.1°, and 5.8°, 5.0° to 6.7°), than in the adaptation blocks (2.5°, 2.2° to 2.7°).

**TABLE 2:** Effect of altitude exposure, acetazolamide and other predictors on directional error.

Predictors	Adaptation		Immediate recall		Post-sleep recall	
	Estimate (95%CI)	*P*	Estimate (95%CI)	*P*	Estimate (95%CI)	*P*
3100 m vs. 760 m in placebo group	2.5 (2.2 to 2.7)	<0.001	5.3 (4.6 to 6.1)	<0.001	5.8 (5.0 to 6.7)	<0.001
Acetazolamide vs. placebo at 760 m	0.9 (−0.2 to 2.1)	0.105	0.6 (−2.1 to 3.4)	0.648	1.3 (-2.3 to 4.9)	0.489
Interaction 3100 m * Acetazolamide#	−0.3 (−0.6 to 0.1)	0.119	−2.7 (−3.7 to −1.6)	<0.001	−3.1 (−4.3 to −2.0)	<0.001
Age, per 1 year increase	−0.1 (−0.1 to 0.0)	0.111	−0.2 (−0.4 to 0.0)	0.072	−0.1 (−0.4 to 0.1)	0.394
Sex, female vs. male	−1.5 (−3.1 to 0.2)	0.079	−2.0 (−6.0 to 1.9)	0.310	−0.6 (−5.8 to 4.7)	0.831
Valid observations, per 1% increase	−0.3 (−0.3 to -0.3)	<0.001	−0.4 (−0.4 to −0.4)	<0.001	−0.4 (−0.4 to −0.4)	<0.001
Intercept	38.0 (33.3 to 42.7)	<0.001	57.9 (46.6 to 69.2)	<0.001	55.0 (40.0 to 69.9)	<0.001
R^2^	0.09	<0.001	0.16	<0.001	0.15	<0.001
Number of observations	40,413		8,081		7,541	

Panel data multivariate linear regression model with patient number defined as panel variable, movement number as time variable; altitude, drug group and sex are binary variables. A positive value indicates an increase in the dependent variable, the directional error (unit degrees). CI denotes confidence interval. The tests at 760 m were performed before starting the study drug. # this term represents the effect of acetazolamide at 3100 m

The group receiving acetazolamide showed a mitigated DE during immediate (−2.7°, −3.7° to −1.6°) and post-sleep (−3.1°, −4.3° to −2.0°) recall blocks compared to placebo at high altitude (*p* < 0.001, both instances). The difference in adaptation performance between the two groups at high altitude was not significant.

When replacing the interaction altitude*drug by SpO_2_*drug, with the binary SpO_2_ variable (upper half and lower half of SpO_2_ data), significant (*p* < 0.001) dependency of DE from SpO_2_ was demonstrated with similar values to that in the first analysis mentioned (Table 3).

**TABLE 3:** Effect of arterial oxygenation, acetazolamide and other predictors on directional error.

Predictors	Adaptation		Immediate recall		Post-sleep recall	
	Estimate (95%CI)	*P*	Estimate (95%CI)	*P*	Estimate (95%CI)	*P*
SpO_2_ (lower half vs. upper half)	2.4 (2.2 to 2.7)	<0.001	5.3 (4.6 to 6.1)	<0.001	6.0 (5.0 to 6.9)	<0.001
Acetazolamide vs. placebo in upper half of SpO_2_	1.2 (0.1 to 2.4)	0.032	0.5 (−2.3 to 3.3)	0.741	1.8 (−1.9 to 5.5)	0.339
Interaction lower half SpO_2_ * Acetazolamide	−0.9 (−1.3 to −0.5)	<0.001	−2.4 (−3.6 to −1.2)	<0.001	−3.6 (−4.9 to −2.2)	<0.001
Age, per 1 year increase	−0.1 (−0.1 to 0.0)	0.155	−0.2 (−0.3 to 0.0)	0.092	−0.1 (−0.4 to 0.1)	0.387
Sex, female vs. male	−1.3 (−2.9 to 0.4)	0.130	−1.7 (−5.7 to 2.3)	0.409	−0.4 (−5.6 to 4.8)	0.888
Valid observations, per 1% increase	−0.3 (−0.3 to −0.3)	<0.001	−0.4 (−0.4 to −0.4)	<0.001	−0.4 (−0.4 to −0.3)	<0.001
Intercept	37.0 (32.3 to 41.7)	<0.001	57.3 (45.9 to 68.7)	<0.001	54.2 (39.3 to 69.1)	<0.001
R^2^	0.08	<0.001	0.16	<0.001	0.14	<0.001
Number of observations	40,413		8,081		7,541	

Panel data multivariate linear regression model with patient number defined as panel variable and movement number as time variable; CI denotes confidence interval; SpO_2_, pulse oximetry. SpO_2_, drug group and sex are binary variables. A positive value indicates an increase in directional error

Individual percentage of valid movements was a significant predictor (*p* < 0.001) of DE in every analysis, meaning that patients who better followed the test rules (investigators had to exclude fewer movements) had a lower DE.

There was no overnight improvement in recall performance in terms of DE whether at 760 m nor at 3100 m in both groups (Figure 3).

### Secondary outcomes

Reaction time improved at high altitude (*p* < 0.001) (Table 4). Improvement in the placebo group was greater than in the acetazolamide group at high altitude (*p* < 0.001) in immediate and post-sleep recall blocks but remained unaffected in 0° rotation blocks. This regression model reveals a significant effect of age on reaction time.

**TABLE 4:** Effect of altitude exposure, acetazolamide and other predictors on reaction time.

Predictors	0° Rotation blocks (morning and evening)		Immediate recall		Post-sleep recall	
	Estimate (95%CI)	*P*	Estimate (95%CI)	*P*	Estimate (95%CI)	*P*
3100 m vs. 760 m in placebo group	−18.9 (−22 to −15.7)	<0.001	−30.3 (−38.6 to −22.0)	<0.001	−36.4 (−45.2 to −27.6)	<0.001
Acetazolamide vs. placebo at 760 m	−18.9 (−50.4 to 12.7)	0.241	−35.6 (−74.3 to 3.1)	0.072	−45.7 (−80.6 to −10.9)	0.010
Interaction 3100 m * Acetazolamide	0.0 (−4.3 to 4.3)	0.998	19.7 (8.4 to 31.0)	0.001	15.6 (3.7 to 27.4)	0.010
Age, per 1 year increase	2.9 (0.8 to 5.1)	<0.001	2.4 (−0.3 to 5.0)	0.078	1.9 (−0.4 to 4.3)	0.107
Sex, female vs. male	−14.9 (−60.8 to 31.0)	0.524	−45.9 (−101.8 to 10.1)	0.108	−28.3 (−78.4 to 21.7)	0.267
Valid observations, per 1% increase	−0.8 (−1.0 to −0.6)	<0.001	−2.8 (−3.0 to −2.6)	<0.001	−2.5 (−2.7 to −2.2)	<0.001
Intercept	198.6 (68.1 to 329.0)	0.003	462.6 (303.5 to 621.7)	<0.001	469.9 (326.8 to 613.1)	<0.001
R^2^	0.02	<0.001	0.11	<0.001	0.10	<0.001
Number of observations	21,198		8,081		7,541	

Panel data multivariate linear regression model with patient number defined as panel variable and movement number as time variable; CI denotes confidence interval. Altitude, drug group and sex are binary variables. A negative value indicates an improvement of reaction time

## Discussion

In this randomized double-blinded placebo-controlled trial, lowlanders with COPD staying at 3100 m revealed an impairment of visuomotor performance during adaptation as well as during immediate and post-sleep recall sessions. Acetazolamide treatment did not prevent impairment in adaptation but improved immediate and post-sleep recall of visuomotor skills without evidence of overnight memory consolidation.

We expected that COPD patients would be susceptible to deterioration of cognitive performance at high altitude considering the known cognitive dysfunction associated with the disease in normoxic conditions and the impaired gas exchange associated with COPD that would predispose to pronounced hypoxemia during exposure to hypobaric hypoxia at high altitude (Furian et al., 2018). Our results support the hypothesis that high altitude-induced hypoxia impairs visuomotor learning and recall as the placebo group showed a significant impairment in DE at 3100 m during adaptation and recall blocks compared to 760 m. In accordance with the role of hypoxemia in visuomotor impairment, the increase in DE was associated with low values of SpO_2_ (Table 3). Consistently, in healthy individuals exposed to normo- or hypobaric hypoxia, the PaO_2_ was a key predictor of cognitive function in various domains (McMorris et al., 2017). Previous studies on the effects of acute altitude exposure on cognitive and motor function in COPD patients are scarce and comparisons are hampered by differences in protocols, altitude and cognitive tasks used. In a study performed in 104 lowlanders with mild to moderate COPD staying for 2 days at 3100 m, we did not observe high altitude-induced changes in psychomotor vigilance reaction time although we noted impairment in postural control (Muralt et al., 2018; Furian et al., 2019). In another study in 32 patients with moderate to severe COPD staying for 2 days at 2590 m, psychomotor reaction time and performance in a trail making test were not affected by altitude (Latshang et al., 2019). In contrast, Kourtidou-Papadeli et al. reported an increase in error rate in a flight simulator task performed by 10 patients with mild COPD at a simulated altitude of 2400 m (Kourtidou-Papadeli et al., 2008). Presumably, complex and interactive visuomotor performance tests such as operating a flight simulator or the target tracking task with rotation imposed on cursor trajectories as in the current study may unravel functional impairments that are not captured by more simple tests (Hornbein, 2001; Petrassi et al., 2012). Unfortunately, data from healthy, age-matched individuals studied under similar conditions at high altitude as the COPD patients in the current study and in the cited investigations are not available and it remains therefore uncertain whether COPD patients are similarly or more susceptible to altitude-induced impairments in cognitive performance compared to healthy individuals.

In the current study, both patient groups receiving preventive treatment with acetazolamide or placebo had a similarly reduced performance during adaptation sessions at 3100 m when compared to 760 m. However, acetazolamide mitigated the high altitude-induced impairment of immediate and post-sleep recall in association with improvements in oxygenation. To our knowledge, effects of acetazolamide on motor learning and recall have not been specifically studied, and data on the effects of the drug on various aspects of cognition in healthy individuals and patients with COPD are controversial (Wang et al., 2013; Yang et al., 2013; Collier et al., 2016; Phillips et al., 2017). Acetazolamide might contribute to improved cognitive functions as it enhances systemic and cerebral oxygenation measured by near infrared spectroscopy in patients with obstructive sleep apnea syndrome during a stay at high altitude (2590 m) (Ulrich et al., 2014). Improvements in cerebral tissue oxygenation by acetazolamide have also been documented by magnetic resonance imaging in healthy individuals during acute, normobaric hypoxia (Wang et al., 2015). However, in 9 hypoxemic COPD patients treated at lowland with acetazolamide for several days, no relevant changes in tests of attention were found even though the treatment had increased the PaO_2_ (Vos et al., 1995). In healthy individuals travelling by air from lowland to 3561 m, a randomized, placebo-controlled trial suggested a reduction in neuropsychological measures of concentration, cognitive processing speed, reaction time and memory by acetazolamide compared to placebo (Wang et al., 2013). A mechanism proposed to explain how acetazolamide may impair cognitive performance in terms of learning and memory function relates to its inhibition of brain carbonic anhydrase, that plays an important role in cerebral signal processing, synaptic transformation and gaiting of memory storage (Sun and Alkon, 2002). Presumably, there might be a trade-off between beneficial effects of acetazolamide on cerebral functions due to improved oxygenation and negative effects by inhibition of carbonic anhydrase specifically in the brain. Whether the net effect of acetazolamide on cognitive performance is positive or negative might depend on the drug dose [with higher doses associated with more pronounced cerebral adverse effects (Collier et al., 2016)], the domain of cognition tested, the degree of hypoxia, age and other, unknown factors.

Our data suggest that overnight motor memory consolidation did not occur in the COPD patients, not even at lowland since DE during post-sleep recall was not reduced in comparison to immediate recall in the evening sessions. Based on electro-encephalographic recordings, it was suggested that slow wave activity during sleep may play a role in visuomotor memory consolidation (Huber et al., 2004; Landsness et al., 2009). Thus, in a previous study (Tesler et al., 2015), in young, healthy lowlanders, an overnight memory consolidation existing at low altitude (490 m) was lost at 2590 m in association with a reduction in slow wave activity during sleep, while adaptation and immediate (pre-sleep) recall of a visuomotor task were unaffected by altitude. A reduction in motor memory plasticity in older patients with COPD could be a possible explanation for impaired overnight motor memory consolidation in COPD patients observed in the current study (Voelcker-Rehage, 2008; Taki et al., 2013; Chen et al., 2016).

At a first glance, the finding of an improved reaction time at high altitude was surprising. However, it is consistent with our observations in a previous study in patients with COPD who showed a reduced psychomotor vigilance reaction time at 2590 m (Latshang et al., 2019). In both studies, a learning effect overriding the effects of hypoxia could not be excluded. Nevertheless, reaction time could have been improved in hypoxic condition because of an enhanced alertness due to an increased sympathetic activity (Wolfel et al., 1994; Tank and Lee Wong, 2015; Nanduri et al., 2019). In the main trial of this project (Furian et al., 2022), an increased mean arterial pressure has been observed in the placebo group compared to the acetazolamide group, as a possible hint for increased sympathetic activity. Acetazolamide did not affect reaction time during blocks with 0° rotation. But reaction time was delayed in the acetazolamide group compared to placebo during the more difficult recall blocks (60° rotation). As mentioned above, altered signal processing in the brain by carbonic anhydrase inhibition may have hampered performance during more difficult visuomotor tasks.

### Limitations

The study design starting with low altitude baseline measurements followed by subsequent high altitude exposure might have resulted in a learning effect on visuomotor performance that counteracted adverse effects of high altitude thereby leading to underestimation of performance decrements in hypoxia. However, we specifically selected a randomized, placebo-controlled parallel design to appraise effects of acetazolamide. As we did not record sleep EEG, we were unable to evaluate whether the absence of overnight memory consolidation was related to reduced slow wave activity in participants taking acetazolamide or placebo. We could not conclusively analyze the potential effect of acute mountain sickness on visuomotor performance because COPD patients who suffered from acute mountain sickness were unable to undergo MTM tests or received oxygen therapy (Furian et al., 2022). Although our data demonstrate significant effects of high altitude and of acetazolamide on performance in an established visuomotor task, further studies are needed to investigate how these findings might translate into clinical outcomes and whether other doses of acetazolamide or different compounds may be more effective.

## Conclusion

The current randomized, placebo-controlled study in patients with moderate to severe COPD indicates that visuomotor performance and memory recall is affected by hypoxia at high altitude. Acetazolamide mitigated these adverse effects of high altitude and might therefore be beneficial for COPD patients performing complex visuomotor tasks during a stay at high altitude.

## Data Availability

The raw data supporting the conclusions of this article will be made available by the authors, without undue reservation.
